# Combining Oxidative Stress Markers and Expression of Surfactant Protein A in Lungs in the Diagnosis of Seawater Drowning

**DOI:** 10.3390/life13010159

**Published:** 2023-01-05

**Authors:** Isabel Legaz, Estefanía Barrera-Pérez, Agustín Sibón, Francisco Martínez-Díaz, María D. Pérez-Cárceles

**Affiliations:** 1 Department of Legal and Forensic Medicine, Biomedical Research Institute (IMIB), Regional Campus of International Excellence “Campus Mare Nostrum”, Faculty of Medicine, University of Murcia, 30110 Murcia, Spain; 2 Institute of Legal Medicine and Forensic Science, 11010 Cádiz, Spain; 3 Anatomical Pathology Service, Reina Sofia University Hospital, 30003 Murcia, Spain

**Keywords:** malondialdehyde, surfactant protein A, glutathione, seawater drowning, forensic pathology

## Abstract

Background and Objectives. The diagnosis of seawater drowning (SWD) remains one of the most complex and contentious. It is one of the leading causes of unintentional death around the world. In most cases, the forensic pathologist must reach an accurate diagnosis from the autopsy findings and a series of complementary tests such as histopathological, biological, and chemical studies. Despite the lung being the most affected organ in death by submersion, there are few studies on this type of death’s impact on this organ. The aim was to investigate human lung cadavers of forensic cases due to different causes of death, the concentration of the oxidative stress markers malondialdehyde (MDA) and γ-glutamyl-l-cysteinyl glycine (GSH), and the relationship with the expression of surfactant protein A (SP-A) to try to discriminate SWD from other types of causes of death. Materials and Methods. A total of 93 forensic autopsy cases were analyzed. Deaths were classified into three major groups based on the scene, cause of death, and autopsy findings (external foam, frothy fluid in airways, overlapping medial edges of the lungs): (a) drowning in seawater (n = 35), (b) other asphyxia (n = 33), such as hangings (n = 23), suffocations (n = 6), and strangulation (n = 4), and (c) other causes (n = 25), such as multiple suffocations. Oxidative stress markers (MDA and GSH) and the immunohistochemical expression of SP-A were determined in both lungs. Results. MDA levels were statistically higher in both lungs in cases of SWD than in other causes of death (*p* = 0.023). Similarly, significantly higher levels of GSH were observed in SWD compared to the rest of the deaths (*p* = 0.002), which was more significant in the right lung. Higher immunohistochemical expression of SP-A was obtained in the cases of SWD than in the other causes of death, with higher levels in both lungs. The correlation analysis between the levels of oxidative stress (MDA and GSH) in the lung tissue and the expression level of SP-A showed positive and significant results in SWD, both in the alveolar membrane and the alveolar space. Conclusions. Determining the levels of MDA and GSH in lung tissue and the expression level of SP-A can be of great importance in diagnosing SWD and the circumstances of death. A better understanding of the physiology of submersion is essential for its possible repercussions in adopting measures in the approach to patients who have survived a submersion process. It is also necessary for forensic pathology to correctly interpret the events that lead to submersion.

## 1. Introduction

The diagnosis of seawater drowning (SWD) remains one of the most complex and controversial deaths and represents one of the leading causes of unintentional death worldwide [1,2,3]. Drowning is appropriately defined as submerging or immersing oneself in a liquid that causes breathing collapse [4]. The precise physiological processes of this process are complex and mostly unknown and have only been recognized speculatively in recent drowning reviews and research [5,6,7,8]. In most cases, the forensic pathologist must reach an accurate diagnosis from the autopsy findings and a series of complementary tests such as histopathological, biological, and chemical studies [9,10,11]. Despite the lung being the most affected organ in death by submersion, there is little information on this type of death’s impact on this organ. When morphological drowning symptoms are absent, it is difficult to determine whether the person died due to drowning or if the body is in an advanced putrefaction stage [12,13,14].

Numerous studies were conducted to investigate various drowning detection methods, such as diatom analysis and chemical analysis of electrolytes and minerals in the blood as water aspiration markers [15,16,17,18], surfactants and nitrogen in the blood, and compound markers for lung injury, haemodilution, and hypoxia [19,20,21]. Research indicates that hypoxia increases the production of reactive oxygen species (ROS) in the brain [22,23,24,25]. Hypoxia reduces the activity of the cytochrome chain responsible for mitochondrial oxidative phosphorylation, resulting in decreased ATP production and increased ROS [26], as well as a reduction in the activity of the cellular antioxidant system [26], potentially leading to oxidative stress.

Previous studies have analyzed the levels of MDA and GSH in lung and heart tissues in forensic cases with different causes of death [27,28]. On the other hand, their research is primarily concerned with determining whether the length of survival time is related to the extent of lipid peroxidation in lung tissue following such ischemia-reperfusion processes and the diagnosis of myocardial damage. Although the lungs are the organs most affected in drowning deaths and provide the most characteristic signs, there are no studies to our knowledge on the concentrations of oxidative stress markers and their possible relationship with microscopic changes such as the distribution of pulmonary surfactant in SWD or any other cause of death.

On the other hand, SP-A is a collectin that is produced by alveolar type II and Clara cells. It binds to carbohydrate structures on microorganisms, triggering innate immunity effector mechanisms and modulating the inflammatory response in the lung [29].

Some authors [30,31] reported a more granular expression of SP-A in drowning victims. Other authors considered that the immunohistochemical staining pattern of SP-A is useful for differentiating drowning but not for differentiating FWD from SWD [32].

The aim was to investigate the concentration of MDA and GSH in human lung corpses from forensic cases with different causes of death to know the relationship of oxidative stress markers with the histological changes produced by the expression (SP-A) to try to discriminate SWD from other types of causes of death.

## 2. Materials and Methods

### 2.1. Forensic Autopsy Cases

Ninety-three corpses were selected from forensic autopsies performed at Cadiz’s Institute of Forensic Medicine (Spain). Cases from routine medico-legal autopsies were chosen at random. There was no history of chronic lung disease among the participants. Deaths were classified into three major groups based on the scene, cause of death, and autopsy findings (external foam, frothy fluid in airways, overlapping medial edges of the lungs): (a) drowning in seawater (n = 35), (b) other asphyxia (n = 33), such as hangings (n = 23), suffocations (n = 6), and strangulation (n = 4), and (c) other causes (n = 25), such as multiple suffocations. The study examined sociodemographic data (age, gender), the postmortem interval (PMI), the weight of both lungs, the main causes of death, and the types of death. The study followed the Helsinki Declaration, and the protocol was approved by the Murcia University Research Ethics Committee (approval number ID1897/2018) and the Andalusia Regional Department of Justice (Spain).

### 2.2. Sample Preparation

A double-blind study was carried out and the laboratory was unaware of the autopsy results. The lungs were removed in their entirety and weighed. The lung dissection equipment was rinsed with ultra-pure sulfuric, nitric acids, and bi-distilled quartz water. Apical lung samples were collected to avoid postmortem hypostasis from interfering with blood distribution [33]. A titanium knife was used to separate the apical bronchopulmonary segment from the rest of the lungs. The samples were frozen and lyophilized after being processed for 48 h in washed polyethylene vials.

### 2.3. Analysis of Oxidative Stress Markers in the Lung

#### 2.3.1. Determination of Malondialdehyde

After extracting lipids from postmortem lung tissues, the colorimetric determination of malondialdehyde (MDA) was used to estimate lipid peroxidation [34]. To extract the lipids, a 1 g sample of lung tissue was homogenized for 10 min in an ice bath with a chloroform–methanol solution (2:1, *v*/*v*); 0.15 mg of butylated hydroxytoluene (BHT) was added to ensure that no lipid oxidation occurred during the assay [33,34,35]. A 15 min centrifugation followed by homogenization at 1500× *g*. The organic layer was pipetted off in a cold nitrogen stream, washed, and evaporated. The extract was weighed and redissolved in methanol (1 mL/mg) before being mixed with 2.5 volumes of cold 20% (*w*/*v*) trichloroacetic acid (TCA) and one volume of 0.67% thiobarbituric acid (TBA) (*w*/*v*) to precipitate protein. The reaction mixture was heated in boiling water for 10 min, and the pink chromogen was measured using spectrophotometry at 532 nm.

The lipid peroxidation levels of the upper lung lobes and the mean level of lipid peroxidation were examined in each of the three causes of death. MDA levels were measured in nanomoles per gram of tissue.

#### 2.3.2. Determination of Glutathione Levels

Determining glutathione (γ-glutamyl-l-cysteinyl glycine, GSH) concentrations in lung tissue was performed by spectrophotometry using Tietze’s enzymatic method [36]. A low GSH is interpreted as evidence of redox unbalance and weakened reducing power [37] GSH levels were expressed as nmol/g tissue.

#### 2.3.3. Determination of Pulmonary Surfactant Protein A

Lung tissue was immunohistochemically examined to determine the expression of pulmonary surfactant protein A (SP-A). PE-10 (1:100; Dako, Kyoto, Japan) mouse anti-human SPA monoclonal antibody was used [38]. The tissues were incubated for 30 min at room temperature. Immunolabeling was detected using the streptavidin/biotin system (OmniTags kit) and 3,3′-Diaminobenzidine (DAB) (Shandon/Lipshaw/Immunon, Pittsburgh, Penn.) according to the manufacturer’s instructions.

Immunohistochemical expression of SP-A was analyzed according to the two different patterns of expression; (1) staining of the wall and interior membrane alveolar, (2) staining of interalveolar granular deposits [39].

The reactivity of SP-A immunostaining on type II alveolar cells and the alveolar surface was evaluated as follows: (0) negative, (1) weakly positive and broken staining line, (2) positive, (3) strongly positive. Findings in the interalveolar space were also classified into four categories:—negative (0), weakly positive (1), positive with some massive aggregates of stained granules (2), strongly and diffusely positive with massive aggregates of stained granules (3) [39]. The evaluation and classification of the intensity level of the histological findings were evaluated and then agreed upon by two different observers.

### 2.4. Statistical Analysis

Data and results on demographics were entered into a database (Microsoft Access 2.0; Microsoft Corporation, Seattle, WA, USA) and analyzed with SPSS 22.0. (SPSS Software Inc., Chicago, IL, USA).

All information was presented as a mean, standard deviation (SD), or percentage. The median, interquartile range (IQR), maximum (Max), and minimum (Min) values were calculated using descriptive statistical analysis.

The data distribution did not have a normal distribution after verification by the Kolmogorov–Smirnov test. The nonparametric Mann–Whitney U test for two samples and the Kruskal–Wallis test by ranks for multiple samples were used to compare mean values with different data groups. *p*-values less than 0.05 were deemed significant.

Spearman’s correlation analysis was used to analyze the possible correlations between the different trace elements, age, postmortem interval, and lung weight. The correlation between levels of oxidative stress markers and reactivity of immunostaining expression findings was also analyzed.

## 3. Results

### 3.1. Sociodemographic Characteristics According to Types and Causes of Death

A total of 93 forensic cases were analyzed and grouped in this study into three main groups; 37.6% of SWD, 35.5% from other types of asphyxia, and 26.9% from other causes of death (Table 1). A total of 83.9% were men, while 16.1% were women. The mean age of the subjects was 50.0 ± 19.2 years (mean ± SD; range 2–86 years) and 83.9% (n = 78) were men. There were no statistically significant differences in the mean age between the sexes. The mean postmortem interval (PMI) was 21.1 ± 8.5 h (mean ± SD; range 10–48 h). No statistically significant differences were found in the PMI between the three groups of causes of death analyzed (*p* = 0.950).

The mean of the weight of all the cases of the right lung was 666.71 ± 265.68 (mean g ± SD), and of the left lung, 589.74 ± 248.75 (mean g ± SD). It was observed that the mean weight of both lungs was higher in SWD compared to the other types of deaths analyzed. Statistically significant differences in left lung weight (*p* = 0.028) were observed among the three cause-of-death groups.

Furthermore, statistically significant differences were found between the weight of both lungs in deaths due to other asphyxiations (*p* = 0.009) and other causes of death (*p* = 0.008), with the right lung having greater weight in both cases. Regarding the mean weight of the lungs, it was significantly higher in SWD (*p* = 0.042) compared to the other two groups of causes of death analyzed.

### 3.2. Oxidative Stress Markers in Lung Tissue

The concentration of MDA and GSH in each lung was analyzed to determine the concentration of oxidative stress markers in lung tissue. The concentration in both lungs was calculated in the three causes of death analyzed (Table 2).

The analysis of the mean concentration of MDA in the total of the analyzed cohort (n = 93) was, in the right and left lung, 3.799 ± 2.267 nmol/g and 4.012 ± 2.232 nmol/g, respectively, the total mean concentration in both lungs being 3.905 ± 0.798 nmol/g. It was observed that the mean values of MDA, both in the right and in the left lung, were higher in cases of SWD than in deaths due to other types of asphyxia and other causes of death. However, there were no statistically significant differences in any case. In contrast, statistically significant differences were found in the mean concentrations of MDA between the three groups of causes of death (*p* = 0.042). The MDA mean concentrations in SWD deaths were significantly higher than other causes of death (4.253 ± 2.012 nmol/g vs. 3.043 ± 1.358 nmol/g; *p* = 0.023); however, these were similar to other types of asphyxia. Mean MDA concentrations in SWD were significantly higher than any other cause of death (4.253 ± 2.012 nmol/g vs. 3.444 ± 1.611 nmol/g; *p* = 0.047).

On the other hand, the GSH analysis showed that the mean concentrations of GSH between the three types of causes of death analyzed presented statistically significant differences (*p* = 0.046), with the concentrations being significantly higher in SWD cases compared to other causes of death (48.841 ± 45.877 nmol/g vs. 16.40 ± 21.17 nmol/g, *p* = 0.030). Overall, GSH concentrations were higher in SWD compared to any other cause of death (48.841 ± 45.877 nmol/g vs. 21.197 ± 23.958 nmol/g; *p* = 0.041).

### 3.3. Immunohistochemical Expression of the SP-A Protein

The immunohistochemical expression of SP-A in histological sections of the upper pulmonary lobe of both lungs was analyzed (Table 3). The results showed a significantly different differential immunohistochemical expression of SP-A in the membrane in the three causes of death analyzed (*p* = 0.000 in both lungs). The highest expression of SP-A was found in cases of death due to drowning compared to other causes of death, both in the right lung (1.862 ± 0.693 vs. 1.072 ± 0.377; *p* < 0.0001) and in the left lung (1.896 ±0.557 vs. 1.109 ± 0.416, *p* < 0.0001).

Regarding the immunostaining results in the alveolar space, they also showed statistically significant differences between the three causes of death in both lungs. It was observed that the expression of SP-A in the alveolar space was significantly higher in SWD cases than any other cause of death, both in the right lung (2.344 ± 0.720 vs. 1.200; *p* < 0.0001) and in the left lung (2.137 ± 0.990 vs. 1.243 ± 0.802; *p* < 0.0001)

Table 4 analyzes the degree of immunostaining corresponding to the expression of SP-A in the upper lobes of both lungs in the three causes of death analyzed. The immunostaining of the alveolar membrane and the alveolar space revealed statistically significant differences between the three groups in both lung lobes. The frequency of intensity of the two and three stainings is significantly higher in cases of death by submersion compared to the other two groups of causes of death. In the control cases, an intensity of three is not observed in any case, the frequency of negative cases being significantly higher, especially in the findings of the alveolar space.

### 3.4. Relationship between Oxidative Stress Levels and the Immunohistochemical Expression of SP-A

A correlation analysis was performed between the concentration of oxidative stress markers (MDA and GSH) and the quantitative level in the expression of protein A of pulmonary surfactant in the upper lobes of both lungs (Table 5). The data showed a positive and significant correlation between the alveolar membrane concentration and alveolar space.

The correlation analysis between the levels of oxidative stress markers (MDA) and the quantitative level in the expression of the SP-A revealed a positive and significant correlation between both, in the upper lobes of both lungs, both in the alveolar membrane, and the accumulation of granules/deposits in the alveolar space. On the other hand, the correlations between the expression of SP-A with the mean concentrations of the antioxidant GSH showed a positive and statistically significant correlation in the immunostaining of the membrane in the right upper lobe (r = 0.315, *p* = 0.007) and the expression of SP-A in the alveolar space in the left upper lobe (r = 0.253, *p* = 0.039).

The analysis of the concentrations in the right or left lung separately showed that the concentrations of GSH were higher in both lungs in the case of SWD compared to other causes of death, finding a statistically significant increase between groups in the right lung and not in the case of the left lung.

**Figure 1:**
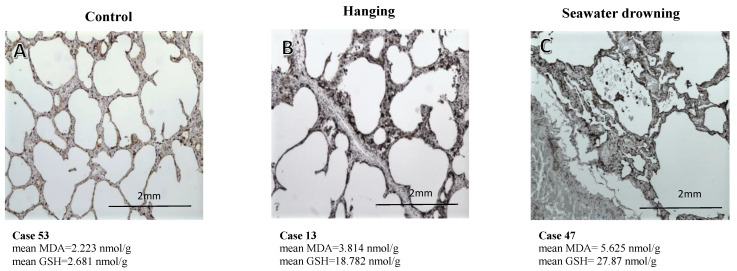
Transversal upper lobe lung sections stained with SP-A in three causes of death: (**A**) Death by a gunshot wound; (**B**) Death by hanging; (**C**) Seawater drowning. The mean concentrations of MDA and GSH were expressed in nmol/g in all cases. (**A**–**C**) with 150× magnification.

Subsequently, the immunoexpression of SP-A in histological sections was analyzed in three different cases: asphyxia due to hanging, SWD and another different cause of death (death by a gunshot wound) as control. The mean concentrations of MDA and GSH were included in each case (Figure 1). The immunoexpression of SP-A in the case of death by a gunshot wound (Figure 1A) showed a weakly positive expression in the membrane with no expression in the alveolar space. The lung tissue, in this case, presented the lowest concentration levels of MDA and GSH. In the case of death by hanging (Figure 1B), a strong immunoexpression of SP-A from the alveolar membrane was observed but without positive findings in the alveolar space. The concentrations of both MDA and GSH were intermediate compared to the SWD case and control case. In the case of SWD, an intense and diffusely positive membrane staining was observed with massive aggregates of stained granules (Figure 1C). The mean MDA and mean GSH concentration in the lung tissue were higher than in the other two cases (mean MDA = 5.625 nmol/g mean GSH = 27.873 nmol/g).

## 4. Discussion

This study analyzed the concentration of oxidative stress markers (MDA and GSH) in human lungs from cadavers and their relationship with the expression of SP-A in different causes of death from forensic cases in order to investigate the processes of oxidative stress in seawater drowning (SWD).

A submersion death’s physiological mechanisms are complex and largely unknown, with many only speculatively described [2,7]. During drowning, the lung is the most vulnerable organ [40]. Forceful ventilatory movements against a closed glottis during laryngospasm can cause mechanical damage. Furthermore, aspirated fluids, both hypertonic and hypotonic, cause changes in pulmonary surfactant and the alveolar–capillary barrier during drowning, leading to systemic hypoxemia [7]. Aspiration of hypertonic seawater draws the fluid from the plasma into the alveoli while also causing surfactant damage. The alveolar–capillary membrane’s integrity is affected by hydrostatic forces [41].

An imbalance between the production of reactive oxygen species and the ability to rapidly detoxify intermediate reagents or repair cell damage causes oxidative stress [42,43]. Moderate oxidation can trigger apoptosis, while if it is very intense, it can cause necrosis [44,45]. Free radicals attack polyunsaturated fatty acids in cell membranes and lipoproteins, transforming them into peroxidized fatty acids that shorten their side chain by releasing malondialdehyde (MDA) [46]. The MDA produced during the breakdown of hyper oxidation products indicates oxidative stress in cells and tissues [47]. On the other hand, GSH is an antioxidant that helps protect cells from reactive oxygen species [48]. Glutathione (GSH) is almost always found in its reduced form.

Cell toxicity is frequently measured by the ratio of reduced glutathione to oxidized glutathione [48].

In our study, the levels of MDA and GSH as indicators of oxidative stress in the upper lung lobes of subjects who had died from different causes of death were determined to test their ability to discriminate between SWD and other causes of death. Furthermore, a positive and significant correlation between MDA and GSH suggests that in the case of deaths with such a short evolution period, there seems to be an increase in oxidizing compounds, especially in deaths due to asphyxiation. However, there is not enough time to allow the consumption of antioxidant substances to keep their concentrations high.

Another study analyzed lipid peroxidation levels in lung tissue in subjects who had died of chest trauma and established a correlation with post-traumatic survival time. The values obtained were closely related to survival time and not chest trauma, with the lowest peroxidation levels in victims without a survival period [28]. These authors found no correlation between the survival period and lipid peroxidation values in the upper lung lobes. In addition, they point to the dying process as a possible variable concluding that the peroxidation phenomenon is related to pulmonary reperfusion in victims who have suffered lung injury.

Lipid peroxidation was also studied in myocardial tissue as a reliable indicator of myocardial damage [27]. They observed higher levels of peroxidation in the group of deceased due to myocardial damage without obtaining significant differences between the subjects who underwent cardiopulmonary resuscitation and those who did not. They also found that the mean value of peroxidation in subjects who died of asphyxiation was below the mean value observed in subjects who died of acute myocardial infarction or chest trauma, perhaps due to decreased oxygen levels in asphyxiation and the attenuation of peroxidation processes. However, analysis at short postmortem intervals did not show a statistically significant correlation in mean lipid peroxidation levels. This means there are no conditions for postmortem peroxidation to proceed without oxygen.

Another study investigated lipid peroxidation levels in the red blood cells and lung tissue of subjects who had undergone radiotherapy, obtaining high blood and lung tissue levels after irradiation [49]. The study focused on the protective action of melatonin against lipid peroxidation, verifying the reduction of MDA levels and establishing it as an effective scavenger of free radicals and a stimulator of antioxidant enzymes within cells.

On the other hand, in our research, we analyzed GSH as an antioxidant marker, verifying statistically significant differences with higher levels of GSH in cases of drowning compared to other causes of death and, in general, higher than any other cause of death. In addition, the concentrations of GSH in the right and left lungs were analyzed separately, which turned out to be higher in both lungs in the cases of submersion in salt water compared to the rest of the groups and were statistically significant in the right lung. In the correlation analysis, we observed a positive and significant correlation between the levels of MDA and GSH.

In our study, we also performed histological analysis on tissue from the upper lung lobes to assess the expression of SP-A. Pulmonary surfactant is a lipoprotein complex that covers the alveolar surface of the lung and is synthesized by type II alveolar epithelial cells. SP-A is the primary pulmonary surfactant [50]. The immunohistochemical expression of SP-A is evidenced in two different patterns: the staining of the membrane of the inner alveolar face and in the alveolar wall and the staining of interalveolar granular deposits [51].

According to our findings, SWD had more immunostaining in the SP-A membrane than the other causes of death. There were no statistically significant differences between the various types of asphyxia and the various causes of death. These results coincide with the studies by Zhu et al. [39], where they revealed a more intense and dense immunostaining of SP-A. Another finding of our research was to obtain significant differences in the immunostaining of the alveolar space of both lungs for the three causes of death, with the expression of SP-A being higher in the cases of SWD.

In another study, the immunohistochemical expression of SP-A was carried out in both the upper and lower lung lobes. It obtained differences between drowning and other types of asphyxia and other causes of death [35]. However, statistically, significant differences were only found in the lungs’ upper lobes, which could be due to functional differences (ventilation/perfusion) in the different regions of the lung.

Similarly, another study analyzed SP-A in lung tissue, confirming an increase in SP-A deaths from suffocation, including drowning and lung pathology [52]. They observed that pulmonary surfactant composition and function are altered in acute lung injury cases. Mechanical asphyxiation is characterized by intense and forced breathing, which stimulates the secretion of the pulmonary surfactant, a marker for interpreting death by hypoxia and, thus, death by submersion. According to the authors, an increase in interalveolar granular SP-A staining could indicate the severity and duration of respiratory distress (agony), whereas linear SP-A staining on the interalveolar surface could indicate an increase in surfactant secretion.

Finally, the existence of a correlation between oxidative stress in lung tissue (MDA and GSH levels) and the level of SP-A expression was also analyzed. Our results showed a positive and significant correlation between the concentrations of MDA and GSH in both upper lobes in the alveolar membrane and the alveolar space and the expression of SP-A.

Our immunohistochemical analysis of SP-A in SWD cases showed intense and positive staining with medium concentrations of MDA and GSH compared to the rest of the groups analyzed. In the case of death by asphyxiation by hanging, intense positive staining of SP-A was observed in the alveolar membrane but with intermediate concentrations of MDA and GSH. In gunshot wound deaths, SP-A immunoreactivity was weakly positive in the membrane and negative in the alveolar space, and MDA and GSH levels were low. These findings seem to suggest a connection between oxidative stress processes and changes in the production and distribution of pulmonary surfactants.

To our knowledge, this is the first study that analyzes biochemical markers of oxidative stress in the lung in forensic cases allowing us to better understand the pathophysiology of death by drowning in salt water. Our results indicate that the decrease in oxygen rapidly causes an increase in oxidative stress processes in the generation of oxidants, as is the case with MDA, and in the rapid production of antioxidants in response, including GSH. In the case of rapid deaths with such a short period of evolution, these processes seem to produce an increase in oxidant compounds, especially deaths due to asphyxia. However, the results suggest an insufficient time to allow the consumption of antioxidant substances to keep their high concentrations.

Under physiological conditions, the balance between oxidants and antioxidants favors antioxidants [53]. However, there is no mention of the balance of oxidants and antioxidants after death in the literature (postmortem interval). The oxidant/antioxidant balance in living animal models of injured tissues differs. In damaged tissues of any living animal, oxidant levels increased while antioxidant levels decreased [54,55]. This event is an induced reaction against damage in a particular body area [56].

An animal study analyzes the levels of endogenous parameters of oxidative stress in the liver and its influence on the postmortem interval [57]. These authors observe how superoxide dismutase (SOD), malondialdehyde (MDA), glutathione peroxidase (GST), total glutathione (tGSH) and nitric oxide (NO) were independent predictor parameters of the postmortem interval between 0 and 6 h.

In this sense, in our study, we found a statistically significant correlation between the lung’s antioxidant levels (mean GSH) and the postmortem interval, considering that we are dealing with human samples and a postmortem interval between 7 and 48 h. However, neither our results nor Sener et al.’s [57] indicate that MDA concentrations do not seem adequate to predict the postmortem interval. Increasing the number of investigations in this line is necessary to provide more scientific evidence.

## 5. Conclusions

In conclusion, our data indicate that high concentration values of MDA are observed in SWD deaths and other types of asphyxia compared to the rest of the deaths analyzed in this study. A high concentration of GSH and SP-A could indicate drowning in salt water compared to the rest of the causes of death. A positive and significant correlation is observed between MDA and GSH, and SP-A is both in the alveolar membrane and the alveolar space, which seems to suggest a connection between oxidative stress processes and changes in the production and distribution of pulmonary surfactant. A better understanding of the physiology of drowning is necessary for its potential ramifications in the approach to patients who have survived a submersion process, as well as in forensic pathology for a correct interpretation of the events that lead to death by drowning, the evaluation of medical responsibilities in fatal incidents, and a more accurate analysis of postmortem findings.

## Figures and Tables

**Table 1:** Sociodemographic and lung characteristics in the different causes and types of death analyzed.

Cause and Type of Death	Number of Cases N = 93	Male	Female	PMI (h)	Mean Weight of Both Lungs (g)	Right Lung Weight (g)	Left Lung Weight (g)	
	n (%) 50.05 ± 19.28 (Mean ± SD) *	N = 78 (83.9) n (%) (Mean ± SD)	N = 15 (16.1) n (%) (Mean ± SD)	N = 93, n (%) 21.17 ± 8.56 (Mean ± SD)	N = 93, (Mean ± SD)	N = 93, (Mean ± SD)	N = 93, (Mean ± SD)	*p* **
Seawater drowning (SWD)	35 (37.6) 50.83 ± 18.62	32 (91.4) 50.7 ± 18.7	3 (8.6) 52.0 ± 21.6	22.0 ± 11.6	724.9 ± 285.3	750.3 ± 304.4	699.5 ±276.1	0.074
Other types of asphyxia	33 (35.5) 48.9 ± 20.2	31 (93.9) 48.2 ± 20.6	2 (6.1) 59.0 ± 12.7	20.3 ± 7.6	474.1 ±183.7	510.0 ± 193.7	439.4 ± 176.6	**0.009**
Hanging	23 (24.7) 49.5 ±21.8	22 (95.7) 49.5 ± 22.3	1 (4.3) 50.0	19.5 ± 6.0	464.1 ± 191.6	502.7 ± 204.0	425.5 ± 181.7	**0.011**
Suffocation	6 (6.5) 52.0 ± 19.9	5 (83.3) 48.8 ± 20.4	1 (16.7) 68.0	25.1 ± 12.4	582.2 ± 58.8	627.5 ± 74.2	537.0 ± 67.8	0.180
Strangulation	4 (4.3) 51.2 ± 19.5	4 (100) 51.2 ± 19.5	-	22.0 ± 2.1	515.8 ± 101.5	528.3 ± 107.3	503.4 ± 98.5	0.231
Other types of death	25 (26.9) 50.4 ± 19.6	15 (60.0) 48.2 ± 20.6	10 (40.0) 52.5 ±15.2	19.4 ± 6.1	617.2 ± 253.9	672.7 ± 198.5	561.6 ± 177.1	**0.008**
Multiple trauma	17 (18.3) 53.1 ± 17.6	8 (47.1) 51.2 ± 20.0	9 (52.9) 54.8 ± 14.0	20.2 ± 4.6	637.9 ± 217.4	691.6 ± 232.7	584.1 ± 206.4	**0.028**
Cardiovascular disease	4 (4.3) 37.5 ± 29.5	3 (75.0) 39.6 ± 35.8	1 (25.0) 31	17.0 ±11.2	440.4 ± 99.8	480.0 ± 89.2	400.8 ± 107.0	0.053
Gunshot	4 (4.3) 41.2 ± 15.3	4 (100) 41.2 ± 15.3	-	14.2 ± 6.6	692.9 ± 79.5	745.9 ± 68.9	640.0 ± 89.1	**0.012**

N: Total number of cases of each group; n: number of cases in each group; PMI Postmortem interval (h). * Mean age in years of each group. ** *p*, Comparisons were made between right and left lung weight (Wilcoxon test). The Postmortem interval is expressed in hours. SD; Standard deviation. SWD; Seawater drowning. Mann–Whitney U test was applied to compare age in causes of death. Comparisons were made between the three main causes of death and right lung weight (*p* = 0.062). The Kruskal–Wallis test compared the three main causes of death and left lung weight (*p* = 0.028). *p*-values marked in bold are statistically significant (*p* < 0.05).

**Table 2:** Oxidative stress markers in upper lobe lung tissue in asphyxia and other causes of death.

		Asphyxia							
Oxidative Stress Markers Concentration Median (IQR) Mean ± SD Min–Max *	Total n = 93	SWD n = 35	Other Asphyxias, n = 33	Other Causes of Death n = 25	P1	P2	P3	P4	P5
MDA concentration									
Right lung	3.438 (2.560) 3.799 ± 2.267 0.624–10.379	3.473 (2.542) 3.967 ± 2.243 0.624–10.379	3.811 (2.743) 3.931 ± 2.133 0.781–9.520	2.819 (2.310) 3.366 ± 2.200 0.724–9.882	0.919	0.906	0.705	0.749	0.773
Left lung	3.800 (1.935) 4.012 ± 2.232 0.766–12.607	3.960 (2.183) 4.512 ± 2.254 0.766–1.199	3.774 (3.248) 4.065 ± 2.836 1.519–12.607	3.475 (1.528) 3.303 ± 1.265 0.859–5.151	0.529	0.623	0.438	0.287	0.910
Mean both lungs	3.493 (2.081) 3.905 ± 0.798 0.77–9.98	3.966 (2.251) 4.253 ± 2.012 1.772–9.981	3.436 (3.162) 3.404 ± 1.345 1.302–5.941	2.965 (1.722) 3.043 ± 1.358 0.387–5.423	**0.042**	0.707	**0.023**	0.233	**0.047**
GSH concentration									
Right lung	2.420 (56.556) 31.270 ± 43.679 0.000–159.3	56.350 (85.575) 52.205 ± 49.707 0.000–159.321	7.070 (34.120) 25.784 ± 39.161 0.0–146.854	3.278 (1.238) 4.551 ± 12.033 0.000–40.851	**0.005**	0.257	**0.002**	**0.015**	**0.017**
Left lung	9.31 (68.78) 37.023 ± 45.977 0.000–162.800	36.120 (78.741) 45.477 ± 48.117 0.000–162.811	3.510 (56.60) 33.22 ± 48.68 0.000–157.301	3.965 (61.64) 28.255 ± 38.428 0.000–100.681	0.534	0.494	0.284	0.233	0.314
Mean both lungs	23.420 (50.15) 34.147 ± 40.777 0.000–153.30	41.080 (81.0) 48.841 ± 45.877 0.000–153.30	21.325 (32.97) 29.507 ± 40.18 0.000–150.3	3.942 (30.82) 16.40 ± 21.17 0.000–66.96	**0.046**	0.210	**0.030**	0.233	**0.041**

n, number of individuals in each cause of death; min–max *, minimum and maximum value of concentration; SWD, Seawater drowning; SD, standard deviation; IQR, Interquartile range GSH, γ-glutamyl-l-cysteinyl glycine; MDA, malondialdehyde; * MDA and GSH concentrations were expressed in nmol/g. P1 Comparisons were made using the Kruskal–Wallis test. P2–4, Comparisons were made using the Mann–Whitney test. P1, Comparisons were made between the three types of death. P2, Comparisons were made between SWD and other asphyxiations. P3, Comparisons between SWD and other causes. of death P4, Comparisons were made between other asphyxia and other causes of death. P5, Comparisons were made between SWD and all other types of death; *p*-values marked in bold were statistically significant (*p* < 0.050).

**Table 3:** Immunohistochemical expression of the SP-A protein in lung tissue in asphyxia and other causes of death.

		Asphyxia							
Degree of SP-A Immunostaining (Mean ± SD) * Immunostaining Degree (0–3)	Total N = 93	SWD N = 35	Other Asphyxia N = 33	Other Causes of Death N = 25	P1	P2	P3	P4	P5
Right lung									
SP-A membrane	1.345 ± 0.630 0–3	1.862 ± 0.6930–3	1.062 ± 0.4350–2	1.087 ± 0.2881–2	**0.000**	**0.000**	**0.000**	0.845	**0.000**
SP-A space	1.595 ± 0.9580–3	2.344 ± 0.7201–3	1.656 ± 0.6530–3	0.565 ± 0.5890–2	**0.000**	**0.004**	**0.000**	**0.000**	**0.000**
Left lung									
SP-A membrane	1.381 ± 0.5990–3	1.896 ± 0.5570–3	1.125 ± 0.4910–2	1.087 ± 0.2881–2	**0.000**	**0.000**	**0.000**	0.684	**0.000**
SP-A space	1.523 ± 0.9750–3	2.137 ± 0.9900–3	1.687 ± 0.5351–3	0.521 ± 0.5930–2	**0.000**	**0.007**	**0.000**	**0.000**	**0.000**

n, number of individuals in each cause of death; SWD, Seawater drowning; IQR, Interquartile range; *p*-values marked in bold are statistically significant (*p* < 0.05). GSH, γ-glutamyl-l-cysteinyl glycine; MDA, malondialdehyde; SP-A, surfactant protein A. * Degree of SP-A immunostaining was collected on a scale 0–3 and evaluated as follows: (0) negative, (1) weakly positive and broken staining line, (2) positive, (3) strongly positive. Findings in the interalveolar space were also classified into four categories: (0) negative, (1) weakly positive, (2) positive with some massive aggregates of stained granules, (3) strongly and diffusely positive with massive aggregates of stained granules. P1, Comparisons were made using the Kruskal–Wallis test. P2–5, Comparisons were made using the Mann–Whitney test. P1, Comparisons made between the three types of death. P2, Comparisons between SWD and asphyxia. P3, Comparisons between SWD and other causes. P4, Comparisons between other asphyxia and other causes. P5, Comparisons were made between SWD and the rest of the types of death.

**Table 4:** Frequency of the degree of SP-A immunostaining according to the cause of death and location in both lungs.

		Right Lung		Left Lung	
Causes of Death	Degree of SP-A Immunostaining *	Alveolar Membrane	Alveolar Space	Alveolar Membrane	Alveolar Space
SWD (%)	0	5.9	0	3,4	10.3
	1	8.8	20.6	10.3	10.3
	2	67.6	38.2	79.3	34.5
	3	17.6	41.2	6.9	44.8
Other types of asphyxia (%)	0	6.3	3.1	6.3	0
	1	81.3	34.4	75	34.4
	2	12.5	56.3	18.4	62.5
	3	0	6.3	18.8	3.1
Other types of death (%)	0	0	47.8	0	52.2
	1	91.3	47.8	91.3	43.5
	2	8.7	4.3.4	87	4.3
	3	0	0	0	0
***p***		**<0.0001**	**<0.0001**	**<0.0001**	**<0.0001**

* Degree of SP-A immunostaining in alveolar cells: (0) negative, (1) dashed weakly positive staining line, (2) positive, (3) strongly positive. Degree of SP-A immunostaining in interalveolar space SP-A: (0) negative, (1) weakly positive, (2) positive with some massive aggregates of stained granules, (3) intense and diffusely positive with massive aggregates of stained granules. *p*-values marked in bold are statistically significant (*p* < 0.05).

**Table 5:** Correlation analysis between levels of oxidative stress markers (MDA and GSH) and the quantitative level in the expression of SP-A.

Upper Lobe Lung	MDA r (*p*)	GSH r (*p*)
Right lung		
SP-A alveolar membrane	0.281 (*p* = **0.011**)	0.315 (*p* = **0.007**)
SP-A alveolar space	0.287 (*p* = **0.009**)	0.183 (*p* = 0.124)
Left lung		
SP-A alveolar membrane	0.252 (*p* = **0.027**)	0.151 (*p* = 0.222)
SP-A alveolar space	0.350 (*p* = **0.002**)	0.253 (*p* = **0.039**)

GSH, γ-glutamyl-l-cysteinyl glycine; MDA, Malondialdehyde; *p*-values marked in bold are statistically significant (*p* < 0.05); r, correlation coefficient; SP-A, surfactant protein A.

## Data Availability

Not applicable. As this is judicial autopsy data, no data can be shared with third parties.
